# HDL Remodeling in Severe Bacterial Infection in Children: A Potential Biomarker for Diagnosis and Therapeutic Monitoring

**DOI:** 10.3390/ijms27209300

**Published:** 2026-10-20

**Authors:** Arianna Finizio, Marco Sarno, Chiara Botti, Alice Castaldo, Andrea Catzola, Gustavo Cernera, Gilda Crispino, Giovanna Di Nardo, Monica Gelzo, Lorenza Lepore, Claudia Mandato, Virginia Mirra, Silvia Moggia, Francesco Nunziata, Francesca Orlando, Antonia Pascarella, Luca Pierri, Margherita Rosa, Fabio Savoia, Paolo Siani, Giuseppe Castaldo

**Affiliations:** 1 Department of Molecular Medicine and Medical Biotechnology, University of Naples Federico II, 80131 Naples, Italy; arianna.finizio@unina.it (A.F.); gustavo.cernera@unina.it (G.C.); giuseppe.castaldo@unina.it (G.C.); 2 CEINGE-Advanced Biotechnologies Franco Salvatore, 80145 Naples, Italy; alice.castaldo@unina.it; 3 Chronic Diseases, Hepatology and Nutrition Unit, AORN Santobono-Pausilipon IRCCS, 80129 Naples, Italy; m.sarno@santobonopausilipon.it (M.S.); g.crispino@santobonopausilipon.it (G.C.); c.mandato@santobonopausilipon.it (C.M.); a.pascarella@santobonopausilipon.it (A.P.); m.rosa@santobonopausilipon.it (M.R.); 4 Clinical Pathology Unit, AORN Santobono-Pausilipon IRCCS, 80129 Naples, Italy; c.botti@santobonopausilipon.it; 5 General Pediatrics and Immuno-Rheumatology Unit, AORN Santobono-Pausilipon IRCCS, 80129 Naples, Italy; a.catzola@santobonopausilipon.it (A.C.); g.dinardo2@santobonopausilipon.it (G.D.N.); f.nunziata@santobonopausilipon.it (F.N.); f.orlando@santobonopausilipon.it (F.O.); 6 Neonatal Intensive Care and Neonatology Unit, AORN Santobono-Pausilipon IRCCS, 80129 Naples, Italy; l.lepore@santobonopausilipon.it (L.L.); l.pierri@santobonopausilipon.it (L.P.); 7 Pediatric Pulmonology and Respiratory Sub-Intensive Care Unit, AORN Santobono-Pausilipon IRCCS, 80129 Naples, Italy; v.mirra@santobonopausilipon.it; 8 Pediatric Intensive Care Unit, AORN Santobono-Pausilipon IRCCS, 80129 Naples, Italy; s.moggia@santobonopausilipon.it; 9 Epidemiology, Biostatistics and Childhood Cancer Registry Unit, AORN Santobono-Pausilipon IRCCS, 80122 Naples, Italy; f.savoia@santobonopausilipon.it; 10 Chronic and Multifactorial Diseases Unit, AORN Santobono-Pausilipon IRCCS, 80129 Naples, Italy; siani.paolo@gmail.com

**Keywords:** cholesterol, severe bacterial infection, sepsis, inflammation, biomarkers

## Abstract

Many pediatric patients with severe bacterial infection (SBI) present with a compromised general clinical picture. Early identification of the infectious and inflammatory state can improve clinical outcomes. Although microbiological culture remains the gold standard for diagnosis, its delayed results highlight the need for early biomarkers to improve clinical decision-making and patient outcomes. During infection, the acute phase response alters lipid metabolism, compromising cholesterol efflux. In this study, we characterized the serum lipid profile and HDL subclasses in 63 children with SBI at hospital admission (T0) and discharge (T1), comparing them with 63 age- and sex-matched healthy subjects. At T0, we found a significant reduction in serum total cholesterol, LDL cholesterol, and HDL cholesterol in subjects with SBI compared to controls. Interestingly, at a cut-off value of 41.5 mg/dL, HDL cholesterol discriminated the two groups with a sensitivity of 76% and a specificity of 86% for SBI. At T1, total and LDL cholesterol increased, while HDL cholesterol remained unchanged. Moreover, we observed at T1 a clear remodeling of HDL classes, with a significant decrease in large HDL with pro-inflammatory activity and an increase in the intermediate and small HDL with anti-inflammatory activity, compared to time T0. This study proposes serum HDL cholesterol as a candidate biomarker and highlights the role of HDL cholesterol and its classes in inflammatory and immune regulation in systemic bacterial infection, potentially identifying patients who could benefit from HDL-based therapies.

## 1. Introduction

In the pediatric literature, there is no single universally accepted definition of “severe bacterial infection” (SBI), although the term commonly includes invasive or clinically significant bacterial infections such as febrile urinary tract infection (UTI), bacteremia, bacterial meningitis, pneumonia, osteomyelitis, and septic arthritis [1]. Many pediatric patients with SBI present with compromised general condition and require intensive care, oxygen or ventilatory support, hydration and nutritional therapy, and prolonged antibiotic treatment; this also represents a substantial burden on healthcare resources. For this population, early identification of infectious and inflammatory status may improve outcomes, including shorter hospital stay and reduced need for intensive care [2,3]. Although microbiological culture remains the gold standard for diagnosis, its delayed results highlight the need for reliable early biomarkers to improve clinical decision-making and patient outcomes [4].

Among the available biomarkers, C-reactive protein (CRP) and serum procalcitonin (PCT) are the most used [4,5,6]; however, both present important limitations. In fact, CRP lacks diagnostic specificity, as its levels may increase in a variety of non-infectious inflammatory conditions, and although PCT is a more accurate biomarker for the diagnosis of bacterial infections in hospitalized patients [4], it shows low diagnostic sensitivity and may provide false-positive results in non-infectious states such as severe trauma or due to physiological increases in the neonatal period [7,8].

Cytokines (e.g., IL-6, IL-8) [9] and presepsin [10,11] have also been proposed as potential markers of SBI. These molecules appear rapidly within a few hours of bacterial exposure, but their utility may be limited by short systemic half-lives [9,10,11]. Furthermore, markers of endothelial and platelet activation, such as soluble P-selectin, are associated with microvascular dysfunction and organ damage in severe pediatric sepsis, but they do not allow for distinguishing bacterial etiology from non-bacterial systemic inflammation [12].

In recent years, clinical research has focused on the use of inflammatory multimarker panels [13,14]; specifically, combining CRP and PCT with early pro-inflammatory cytokines such as IL-6 and IL-8 makes it possible to cover different kinetic windows of the host immune response [15]. However, the simultaneous use of different biomarkers with distinct secretion kinetics and half-lives can lead to discrepancies in values, complicating rapid interpretation.

Bacterial infections can cause alterations in lipid metabolism and the lipoprotein profile as part of the host’s inflammatory response [16]. Low circulating low-density lipoprotein (LDL)-cholesterol levels have been associated with increased mortality in patients with sepsis and critical illness, as evidenced by observational studies and recent meta-analyses [17,18]. However, the interpretation of LDL-cholesterol as a potential biomarker in infections is limited by its low specificity, the influence of comorbidities and concomitant treatments, temporal variability associated with the inflammatory response, and—in the presence of hypertriglyceridemia—the potential inaccuracy of the formulas used to estimate it [19,20]. Furthermore, an association between triglyceride levels and clinical outcomes appears less consistent and more strongly influenced by metabolic and nutritional factors [18]. Another potential indicator of interest is lipoprotein(a), given its pro-atherogenic, pro-inflammatory, and pro-thrombotic activity; however, available data regarding acute infections remain limited and inconclusive, with studies reporting variations in lipoprotein(a) levels in relation to inflammatory and thrombotic status, yet showing no clear independent association with mortality [21,22,23].

High-density lipoproteins (HDL) play a central role in inflammation, immune regulation, and in the maintenance of endothelial functions [24]. During infection, the acute-phase response alters lipid metabolism, impairing cholesterol efflux and leading to reduced circulating HDL levels and, consequently, reduced anti-inflammatory activity. Moreover, HDL undergo structural and compositional remodeling, which can shift their functional profile toward a pro-inflammatory phenotype further compromising reverse cholesterol transport, or to an anti-inflammatory phenotype that contributes mitigating the inflammatory status. Indeed, the functional properties of HDL are influenced by its heterogeneous subfractions which differ in size, structure, and molecular composition, and may exert either anti-inflammatory (small and medium HDL classes) or pro-inflammatory effects (large class) [25,26].

Alterations in HDL class distribution with a reduction in small HDL are associated with more severe infections [27] and predict the risk of sepsis [28]. While, in chronic inflammatory conditions such as rheumatoid arthritis, an increase in large HDL and a concomitant reduction in small HDL classes have been observed [29]. Notably, an inverse correlation between CRP and small HDL has been described, suggesting the anti-inflammatory role of small HDL [29]. We also found a decrease in small HDL in COVID-19 vaccine-related inflammation [30] as well as in multisystem inflammatory syndrome in children (MIS-C), a severe post-COVID-19 condition characterized by a delayed hyperinflammatory response to SARS-CoV-2 [31].

In this study, we aimed to investigate the serum lipid profile and HDL classes and subfractions in children with SBI at the time of hospitalization and discharge in comparison with healthy controls. In addition, we studied the correlations of HDL-cholesterol and HDL classes versus inflammation markers to evaluate their pathophysiological role and their potential value as candidate biomarkers in SBI.

## 2. Results

### 2.1. Study Population

**Figure 1:**
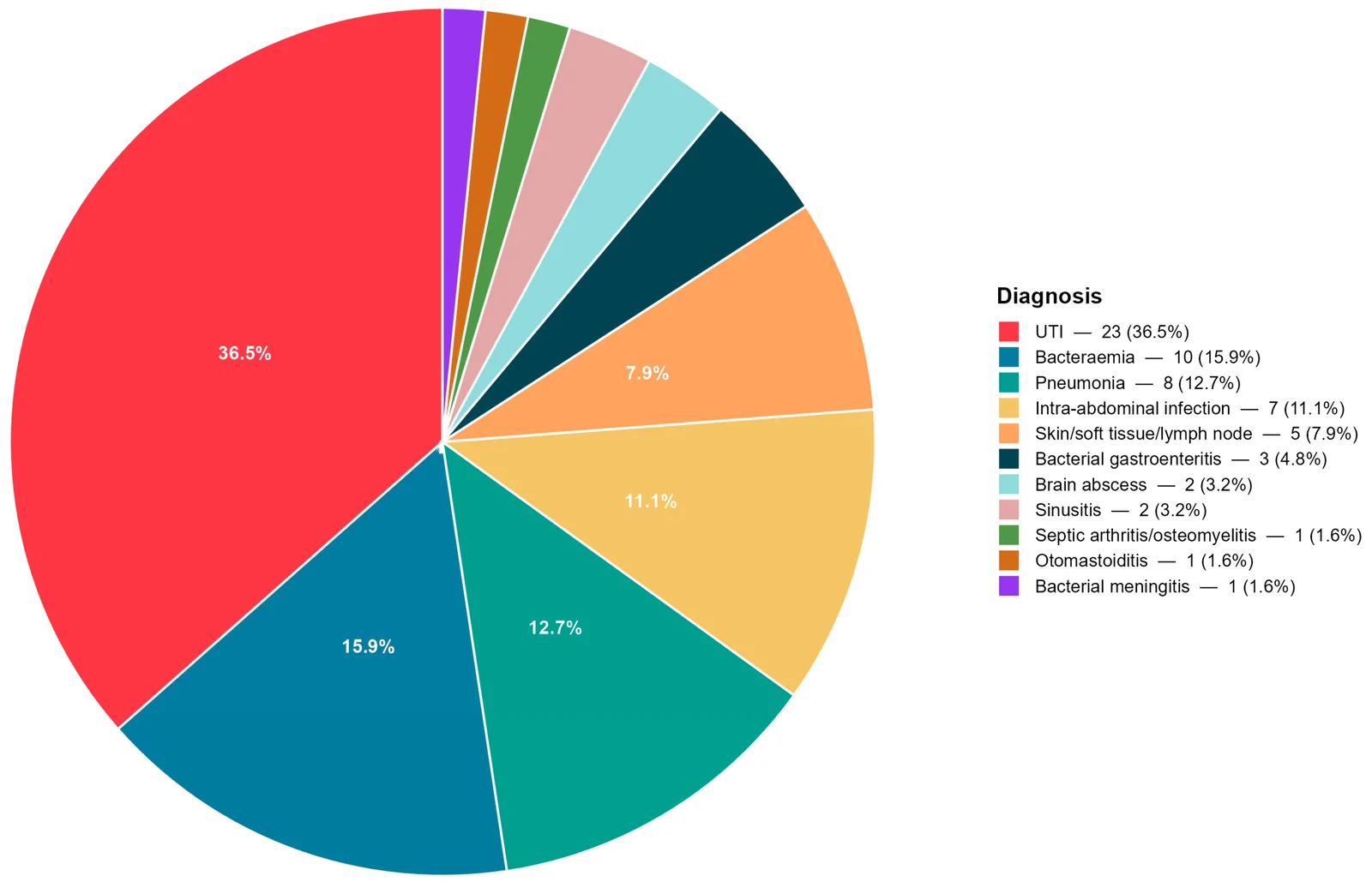
Distribution of severe bacterial infection (SBI, n = 63) diagnoses in the study population. UTI: urinary tract infection.

We enrolled 63 children with SBI (30 females, 47.6%). The median age was six months (interquartile range, IQR: 2–75 months). A control group (CTRL) of 63 subjects (33 females, 52.4%) with a median age of 24 months (IQR: 2–72 months) was also enrolled. No significant differences in age and sex frequencies were observed between children with SBI and CTRL. Study population’s demographic and laboratory data are reported in Table S1. Diagnoses are summarized in Figure 1.

UTI was the most frequent diagnosis (23 cases, 36.5%). Four patients (6.3%) met the diagnostic criteria for sepsis according to the Phoenix Sepsis Score. Nineteen patients (30.2%) had underlying comorbidities. The most common was cerebral palsy (7 cases, 36.8%), followed by urinary or intestinal malformations (6, 31.6%), genetic disorders (4, 21.1%), congenital heart disease (1, 5.3%), metabolic disorders (1, 5.3%), chronic lung disease (1, 5.3%), and inflammatory bowel disease (1, 5.3%). Two patients had multiple comorbidities. Blood cultures were obtained in 57 of 63 cases (90.5%), of which 10 (17.5%) yielded positive results. The most frequently isolated pathogen was *Escherichia coli* (4 isolates, 40%), followed by *Klebsiella pneumoniae* (2, 20%) and *Staphylococcus aureus* (2, 20%). Microbiological isolates are summarized in Table 1.

All patients received broad-spectrum antibiotic therapy, which consisted of combination therapy in 30 cases (47.62%). The median duration of treatment was 10 days (range 6–14.5 days). Intravenous rehydration was required in 45 patients (71.4%), while seven patients (11.1%) received steroid therapy. Respiratory support was required in eight patients (12.7%), including mechanical ventilation in four cases (50%) and high-flow nasal cannula in four cases (50%). One patient (1.6%) required inotropic support. The median length of hospital stay was 10 days (range 7–22.5 days).

Table 2 describes blood cell counts and serum inflammation markers in children with SBI at the time of hospitalization (T0) and discharge (T1). White blood cell (WBC) and neutrophil counts as well as serum CRP and PCT values were elevated at T0 in comparison to reference values in healthy children. The median values of all these biomarkers were significantly lower at T1 compared to T0 and fell within the reference ranges. While, lymphocyte and platelet counts were significantly increased at T1, although the median values were within the reference ranges.

### 2.2. Lipid Profile Characterization

**Figure 2:**
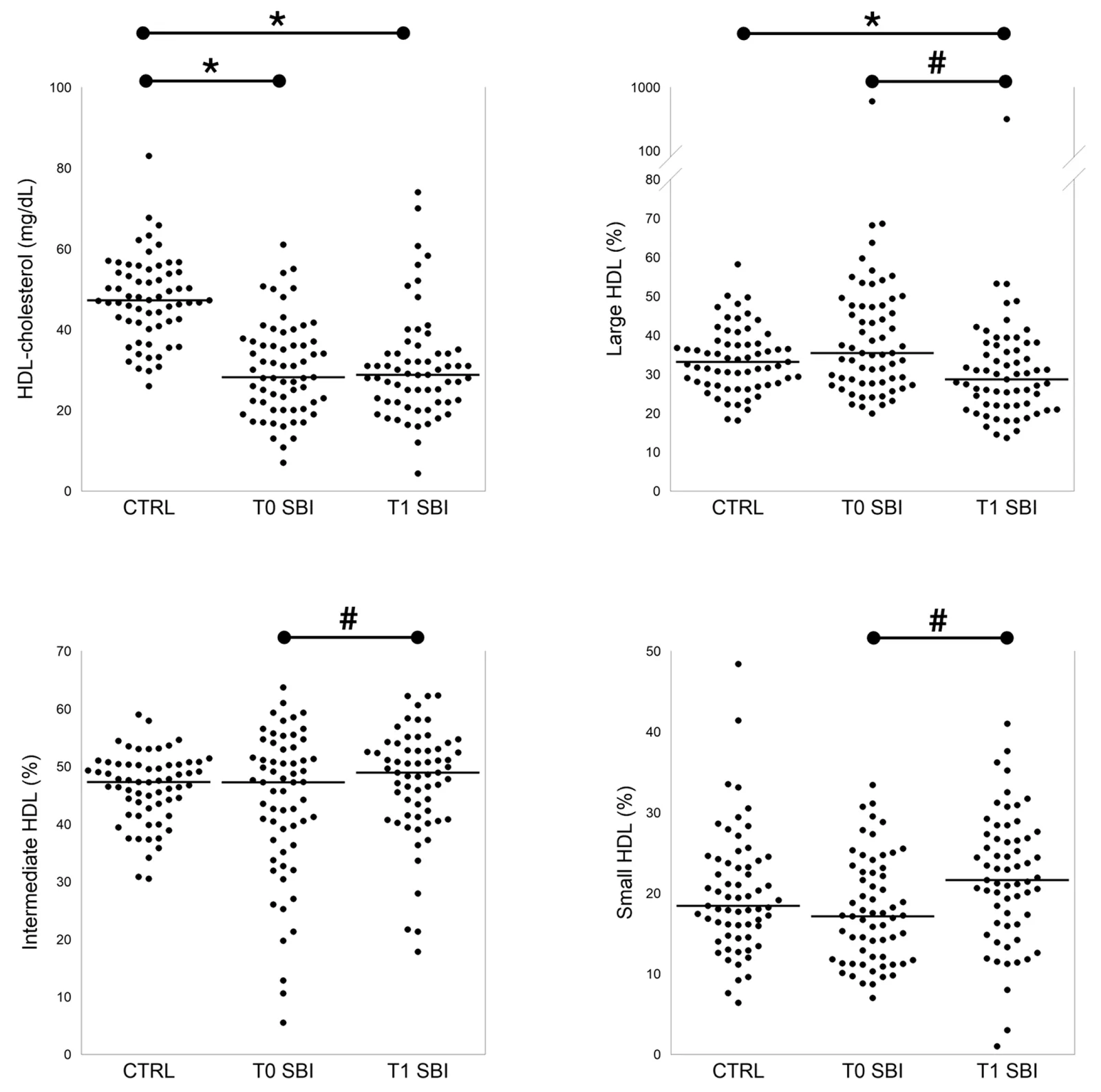
Comparison of HDL-cholesterol and HDL classes among healthy controls (CTRL, n = 63) and severe bacterial infections (SBI, n = 63) at T0 and T1. Black lines in the graphs represent median values. * *p* < 0.05, Mann–Whitney U test; # *p* < 0.05, Wilcoxon test.

We analyzed the lipid profile and HDL subfractions in children with SBI at T0 and T1 in comparison with CTRL (Table 3). In children with SBI at T0, serum total, LDL- and HDL-cholesterol levels (Figure 2) were significantly lower than those in CTRL. At T1, total and LDL-cholesterol levels significantly increased compared to T0 and were comparable to those in CTRL, while HDL-cholesterol levels did not change (Figure 2). Regarding HDL classes, we found an increase in large HDL with lower small HDL in children with SBI at T0 than CTRL (although not significant) (Table 3 and Figure 2).

At T1, large HDL were significantly reduced compared to T0, while intermediate and small HDL were increased (Table 3 and Figure 2). Within the large HDL class, HDL-1 subfraction was significantly higher in children with SBI at T0 than CTRL, and it decreased at T1 as also observed for HDL-2 and HDL-3 subfractions (Table 3). Among the intermediate HDL class, we found significantly lower HDL-7 in children with SBI at T0 than CTRL, and this subfraction together with HDL-6 significantly increased at T1 (Table 3). On the contrary, HDL-4 was reduced at T1 compared to T0 (Table 3). Among the small HDL class, HDL-8 was significantly lower in children with SBI at T0 than CTRL and increased at T1 as also observed for HDL-9 and HDL-10 subfractions (Table 3).

**Figure 3:**
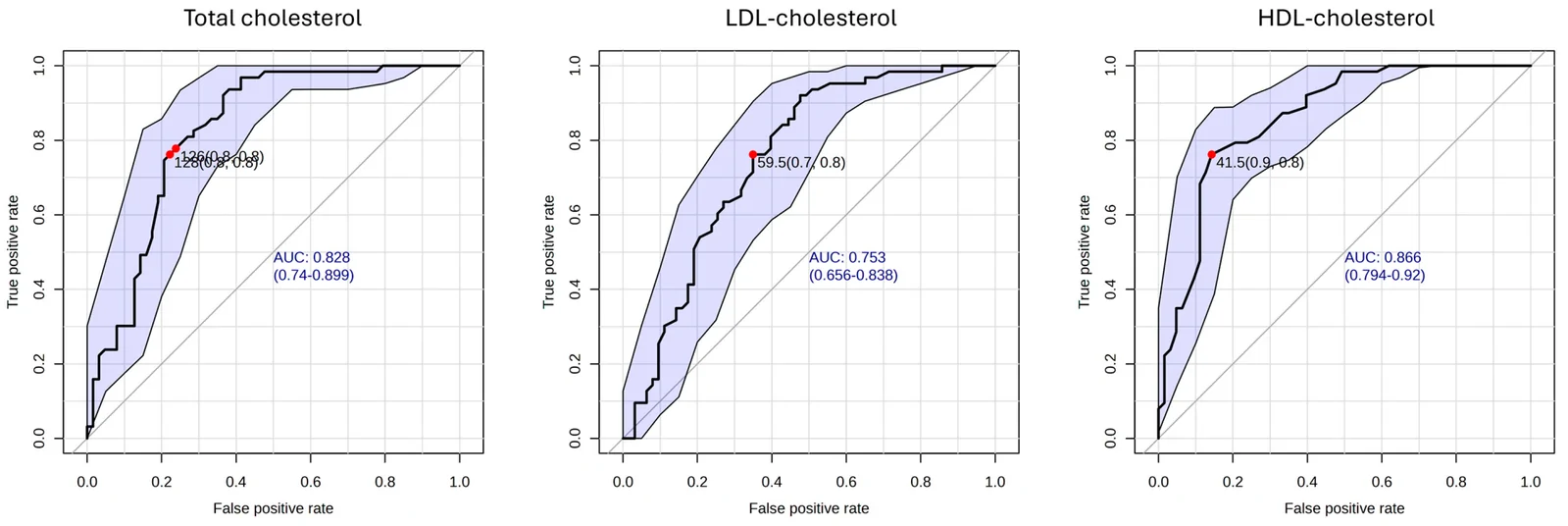
Receiver operating characteristic (ROC) curve analysis of total cholesterol, LDL-cholesterol, and HDL-cholesterol for discrimination between patients with severe bacterial infections (SBI, n = 63) and healthy controls (CTRL, n = 63). The area under the curve (AUC) with 95% confidence interval and the optimal cut-offs (red points) determined according to the “closest to the top-left” criterion are shown.

Then, we applied the receiver operating characteristic (ROC) curve analysis to assess the diagnostic performance of lipid variables (Figure 3). For total cholesterol, the ROC analysis yielded an AUC of 0.828; using the closest-to-the-top-left criterion, two equivalent optimal cut-offs were identified (126.5 and 128 mg/dL), with sensitivities of 77.8% and 76.5% and specificities of 76.5% and 77.8%, respectively. For LDL-cholesterol, the ROC analysis yielded an AUC of 0.753 and identified an optimal cut-off of 59.5 mg/dL with a sensitivity of 76.2% and a specificity of 65.1%. While, for HDL-cholesterol we observed an AUC of 0.866 and an optimal cut-off of 41.5 mg/dL with a sensitivity of 76.2% and a specificity of 85.7%. For HDL classes, the ROC curve analysis showed AUC values lower than 0.600 (Figure S1).

Finally, we performed Spearman’s correlation analysis between all analyzed variables in children with SBI (n = 63) at the time of hospitalization, including patient’s age and body weight (BW) (Table S2). We found significant positive correlations of both age and BW versus total cholesterol, LDL-cholesterol, intermediate HDL, HDL-4, HDL-5 and HDL-6 (*p* < 0.005), and negative correlations of both age and BW versus lymphocyte count, small HDL, HDL-9 and HDL-10 (*p* < 0.008). No significant differences in HDL-cholesterol levels and HDL classes were observed between males and females .

**Figure 4:**
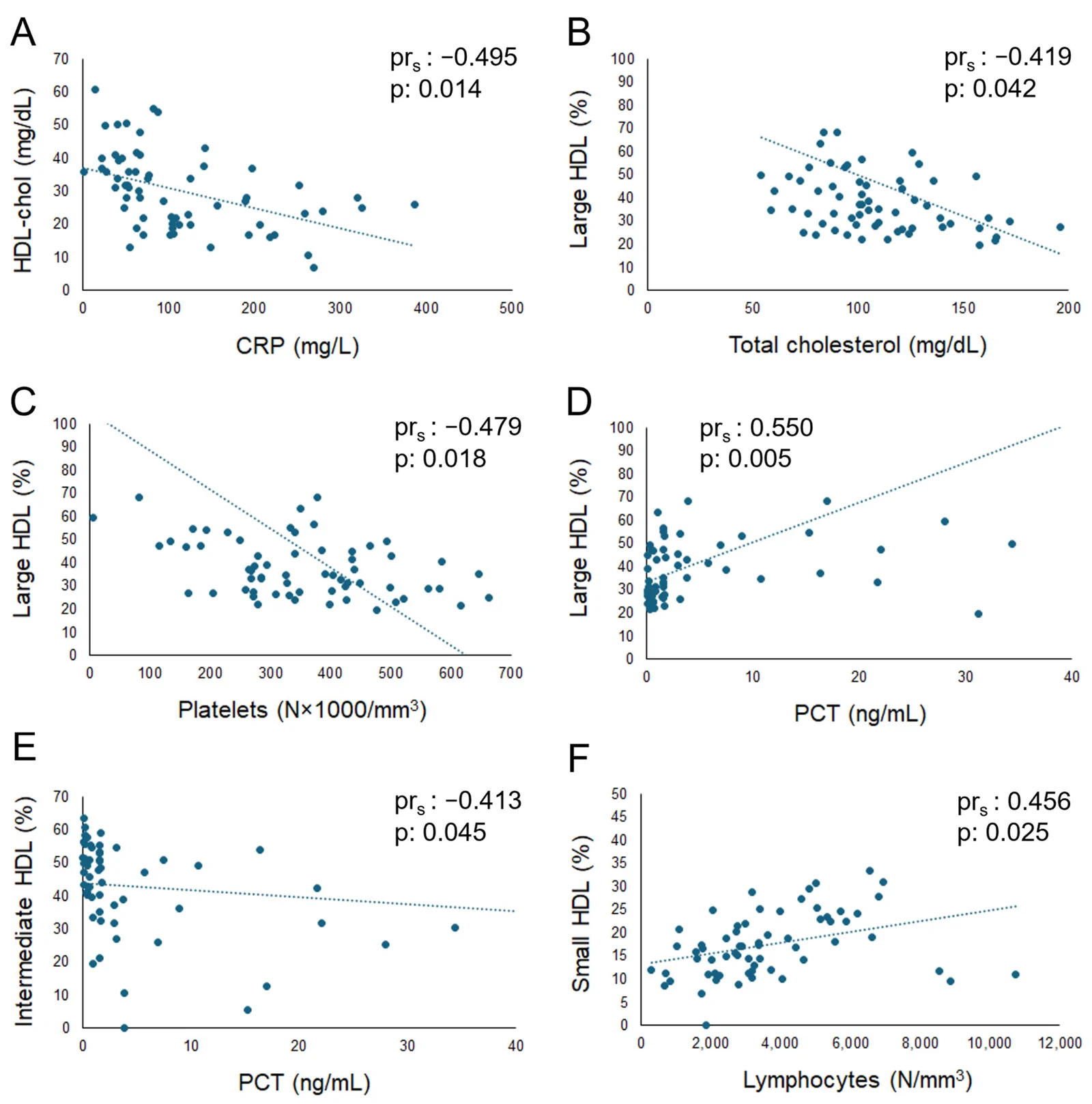
Partial Spearman’s correlation analysis, removing and controlling for the effects of patients’ age and body weight in children with severe bacterial infections (SBI, n = 63) at the time of hospitalization, between (**A**) HDL-cholesterol (HDL-chol) and C-reactive protein (CRP), (**B**) large HDL and total cholesterol, (**C**) large HDL and platelet count, (**D**) large HDL and procalcitonin (PCT), (**E**) intermediate HDL and PCT, and (**F**) small HDL and lymphocyte count. pr_s_: partial Spearman’s correlation coefficient.

Therefore, we applied partial Spearman’s correlation analysis on all analyzed variables removing and controlling the effect of age and BW (Table S3). In particular, we observed a significant negative correlation between HDL-cholesterol and CRP levels (Figure 4A). In addition, large HDL percentages were negatively associated with total cholesterol levels (Figure 4B) and platelet count (Figure 4C), and positively associated with PCT (Figure 4D). On the contrary, intermediate HDL percentages were negatively associated with PCT (Figure 4E). Finally, we found a positive correlation between small HDL and lymphocyte count (Figure 4F).

Regarding the type of infection, Spearman correlation analysis showed a direct correlation between blood culture positivity and large HDL % together with an inverse correlation between blood culture positivity and intermediate HDL % (Table S2). However, these correlations were no longer significant applying partial Spearman correlation removing and controlling for the effects of patient’s age and BW (Table S3).

## 3. Discussion

We characterized serum lipid profile and HDL subfractions in 63 children with SBI at hospital admission (T0) and discharge (T1) in comparison with 63 healthy age- and sex-matched controls. At T0, we found a significant reduction in total cholesterol, LDL- and HDL-cholesterol compared to controls. These results agree with previous findings in both adults and children with severe infectious diseases and sepsis [34] and in children with a severe post-COVID-19 condition, i.e., MIS-C [31]. Furthermore, we found a significant negative correlation between HDL-cholesterol and the inflammatory marker, CRP. These findings support the hypothesis that HDL-cholesterol is directly involved in the inflammatory process. The observed reduction in HDL-cholesterol levels may reflect the profound metabolic adaptations that occur in SBI. In fact, the acute-phase response impairs cholesterol efflux through the action of inflammatory proteins [24]. Interestingly, at T0 using the cut-off of 41.5 mg/dL HDL cholesterol showed a diagnostic sensitivity of 76% and a diagnostic specificity of 86% for the diagnosis of SBI. Such figures appear relevant when compared to the same parameters of CRP (diagnostic sensitivity: 84% and diagnostic specificity: 75%) and PCT (diagnostic sensitivity: 79% and diagnostic specificity: 85%) evaluated by Andreola et al. in 94 children with SBI and 314 non-SBI children [35]. At T1, total and LDL-cholesterol increased and were comparable to those in CTRL, but HDL-cholesterol did not change. We therefore extended the study to HDL subfractions.

At T0, although HDL-cholesterol concentrations were reduced, we observed an increasing trend in the percentage of large HDL class, with pro-inflammatory activity [25], and a decreasing trend in the percentage of small HDL class, with anti-inflammatory activity [36], compared to healthy controls. Furthermore, the increasing trend of large HDL was associated with the reduction in total cholesterol. In agreement with the known pro-inflammatory activity, we found a significant positive correlation between large HDL class and PCT. Furthermore, we observed a negative correlation between large HDL and platelets, which are consumed in SBI mainly due to systemic activation and are thus reported as novel mediators of the inflammatory cascade [37]. On the other hand, the association between small HDL class and the lymphocyte count alone suggests a relationship with acquired immunity, or at least a probable role in specific immune processes involving lymphocytes, rather than in systemic inflammation as a whole.

Meanwhile, at T1, we observed a clear remodeling of HDL subfractions with a significant decrease in large HDL class percentage and an increase in intermediate and small HDL class percentages compared to T0. In agreement, we previously found a decrease in intermediate and small HDL classes in the acute phase of MIS-C together with an inverse correlation between these HDL classes and inflammation markers [31]. Small HDL represents the native HDL subfractions that are converted into intermediate and large HDL [38]. Large HDL are deprived of cholesteryl esters and enriched with unesterified cholesterol, triglycerides and free fatty acids [39]. This conversion represents an acute-phase response, and it was previously described in infectious diseases related to disease morbidity and mortality [27], and in acute COVID-19 [36].

Although the true role of the intermediate HDL class is not yet fully understood, we observed at T1 a significant decrease in the HDL-4 subfraction percentage, which in terms of size is closer to the large HDL class, with a significant increase in the HDL-6 and HDL-7 subfractions, closer to the small HDL class, compared to T0. This indicates the coexistence within the intermediate HDL class of subfractions with pro- and anti-inflammatory activity, although with the balance shifted toward anti-inflammatory activity. This hypothesis is supported by the results of Spearman’s correlation analysis which showed significant negative correlations between the intermediate HDL and PCT.

Finally, we noted that despite the clear remodeling of HDL toward an anti-inflammatory response, at hospital discharge, HDL-cholesterol concentrations were still low and not significantly different from the acute phase. Recovery of total HDL-cholesterol levels likely represents a later event. It would have been useful to reassess HDL-cholesterol and its subfractions at a potential follow-up after hospital discharge, and this represents a study limitation. Although we found significant differences among the variables, strong correlations were not observed between them, with the stronger correlation coefficient equal to 0.550 between large HDL and PCT. However, the associations found between plasma HDL subfraction levels and systemic inflammatory processes open the way to select patients to be addressed to potential therapeutic strategies aimed at both increasing HDL levels [40] and modulating its subfractions [41].

Other study limitations include the limited number of SBI patients enrolled and the heterogeneity of the various severe bacterial infections, further complicated by comorbidities. In addition, in-hospital interventions, particularly intravenous fluid administration, may have influenced circulating lipid concentrations through hemodilution and therefore represent a potential confounding factor. The relatively limited sample size, determined by the number of eligible children with SBI recruited during the study period, may have limited the statistical power to detect smaller between-group differences and may affect the generalizability of the findings. Furthermore, it is appropriate to evaluate in a subsequent study HDL-cholesterol power to discriminate SBI from other non-infectious inflammatory diseases and/or severe non-bacterial infections. A methodologically distinct limitation concerns the choice of the comparison group; our findings on the discriminant ability of HDL-cholesterol should therefore be regarded as hypothesis-generating and warrant validation in prospective cohorts recruited at the point of clinical suspicion. Finally, the lack of cytokine and serum amyloid A measurements limits our ability to directly investigate the mechanisms underlying HDL remodeling during the acute-phase response.

## 4. Materials and Methods

### 4.1. Study Design and Participants

We conducted a prospective study (2024–2025) involving 63 pediatric patients diagnosed with SBI who were admitted to the Chronic Disease, Hepatology and Nutrition Unit, Immuno-Rheumatology Unit, Neonatal Intensive Care Unit, and Pediatric Intensive Care Unit of Santobono-Pausilipon Children’s Hospital. The study was approved by the Ethics Committee Campania 1 (Prot. 27/24 OSS SP 15/11/24, 2/25 OSS E 06/16/25, 1/26 OSS 02/26/26). Healthy age- and sex-matched CTRL (n = 63) were recruited during routine outpatient evaluations and follow-up visits. All CTRL underwent clinical and laboratory examinations to rule out inflammatory/infectious diseases and other pathological conditions.

Written informed consent to participate in this study was provided by the legal guardian/next relative of both SBI and CTRL.

Exclusion criteria included a diagnosis of a genetic or metabolic disorder affecting lipid metabolism (e.g., familial hypercholesterolemia), total parenteral nutrition (for reasons other than the acute infectious event), and refusal or inability to provide informed consent.

For each enrolled patient, we collected demographic and laboratory data (Table S1). We used PCT and CRP cut-off values > 0.5 ng/mL and >20 mg/L, respectively, as these thresholds are considered optimal for identifying invasive and serious bacterial infections [32,33]. Diagnoses were established in accordance with current recommendations and best practice guidelines available in the literature [42,43,44,45,46,47,48,49,50,51,52,53,54,55,56]. Sepsis and septic shock were defined according to the Phoenix Sepsis Score criteria [57]. Bacteremia was defined as a positive blood culture yielding a true pathogen other than CoNS or other commensal bacteria, which were considered contaminants unless documented and treated as true infections [58].

No formal a priori sample size or power calculation was performed. The study included all consecutive children with SBI who met the predefined inclusion criteria during the study period. Thus, the final sample size was determined by the number of eligible patients available for recruitment during the study period.

### 4.2. Biochemical Analyses

Blood samples were collected in appropriate vacuum tubes and processed according to standard laboratory protocols. A complete blood count was performed on EDTA-anticoagulated whole blood using the Sysmex XN-1000 automated hematology analyzer (Sysmex Corporation, Kobe, Japan). Total cholesterol, HDL- and low-density lipoproteins (LDL)-cholesterol were evaluated in serum by Cobas 6000 analytical platform (Roche Diagnostics, Basel, Switzerland) using specific commercial kits (Roche Diagnostics). CRP and PCT were determined on plasma samples obtained from lithium-heparin tubes. Specifically, CRP was quantified by an immunoturbidimetric assay using the Roche Tina-quant CRP4 reagent kit (Roche Diagnostics), while PCT was measured using an electrochemiluminescence immunoassay (Roche Elecsys PCT, ECLIA, Roche Diagnostics). All assays were performed in strict accordance with the manufacturer’s instructions. Instrument calibration, internal quality control procedures, and external quality assessment programs were regularly executed to ensure analytical accuracy and reproducibility.

### 4.3. HDL Subfraction Analysis

The analysis of HDL subfractions was performed by the Quantimetrix kit (Quantimetrix Corp., Redondo Beach, CA, USA). In brief, serum (25 μL) was added to the precast high-resolution polyacrylamide gel tubes along with Lipoprint HDL Loading Gel solution (300 μL). The tubes were photopolymerized and subjected to electrophoresis. Then, the tubes were scanned and analyzed using the Lipoprint LDL/HDL subfractions system. The HDL were separated on the basis of their size into 10 subfractions, which are subsequently grouped into three main classes: large (from HDL-1 to HDL-3), intermediate (from HDL-4 to HDL-7), and small (from HDL-8 to HDL-10) [30,31]. The values of classes and subfractions were reported as % of total HDL.

### 4.4. Statistical Analysis

Continuous data were reported as median (IQR). Categorical data were reported as counts and percentages. The Shapiro–Wilk test was applied to evaluate the normality of the distributions. Paired comparisons were evaluated using the Wilcoxon test. Comparisons between two groups of independent samples were evaluated by the Mann–Whitney U test. The correlations between variables were assessed using Spearman correlation analysis. Partial Spearman correlation analysis was applied to remove and control the effect of age and BW. Univariate ROC curve analysis was performed to assess the diagnostic performance of lipid variables. The area under the curve (AUC) with 95% confidence intervals was calculated, and the optimal cut-off value was determined according to the “closest to the top-left” criterion, with the corresponding sensitivity and specificity reported. Statistical analysis was performed by SPSS (version 30, IBM SPSS Statistics) and MetaboAnalyst 6.0 online package [https://www.metaboanalyst.ca, (accessed on 1 June 2026)]. Graphics were performed by KaleidaGraph software (version 4.5.4, Synergy, Reading) and with R (version 4.5.1) using the ggplot2 package. *p* values  <  0.05 were considered significant.

## 5. Conclusions

In conclusion, our findings indicate that SBI in children is associated with marked alterations in HDL-cholesterol levels and HDL class percentages. Reduced HDL-cholesterol at hospital admission, together with its inverse association with CRP and its diagnostic performance in distinguishing children with SBI from CTRL, supports its potential role as an early candidate biomarker. Moreover, the shift from a higher proportion of large HDL toward intermediate and small HDL classes during clinical recovery suggests that HDL remodeling may reflect the dynamic response to systemic inflammation. These findings highlight the potential relevance of HDL composition, beyond HDL-cholesterol levels alone, in monitoring inflammatory and metabolic changes during SBI. Further prospective studies including appropriate non-bacterial inflammatory controls and larger pediatric cohorts are warranted to validate the diagnostic and monitoring value of HDL-cholesterol and its classes, and to explore their potential relevance for HDL-targeted therapeutic strategies.

## Figures and Tables

**Table 1:** Microbiological investigations and culture results in 63 children with SBI.

Microbiological Investigations	n	%
Blood cultures performed, n (%)	57/63	(90.5%)
- Positive, n (% of performed)	10/57	(17.5%)
- *Escherichia coli*, n	4/10	
- *Klebsiella pneumoniae*, n	2/10	
- *Staphylococcus aureus*, n	2/10	
- CoNS, n	1/10	
- *Streptococcus agalactiae*, n	1/10	
Urine cultures performed, n (%)	40/63	(63.5%)
- Positive, n (% of performed)	25/40	(62.5%)
- *Escherichia coli* (*E. coli*), n	20/25	
- *Klebsiella pneumoniae*, n	2/25	
- *Citrobacter freundii*, n	1/25	
- *Proteus* spp., n	1/25	
- *Pseudomonas aeruginosa*, n	1/25	
Lumbar punctures performed, n (%)	4/63	(6.3%)
- Positive, n (% of performed)	1/4	(25.0%)
- *Streptococcus pyogenes*, n	1/1	
Stool cultures performed, n (%)	12/63	(19%)
- Positive, n (% of performed)	3/12	(25%)
- *Campylobacter jejuni*, n	1/3	
- Shiga toxin-producing *E. coli*, n	1/3	
- *Salmonella* spp., n	1/3	

SBI: severe bacterial infection; CoNS: Coagulase-Negative Staphylococci.

**Table 2:** Comparison of blood cell counts and serum inflammation markers in children with SBI (n = 63) at the time of hospitalization (T0) and discharge (T1).

Markers	Reference Values	T0	T1
WBC (N/mm^3^)	5700–13,500 ^a^	14,315 (7375–19,815)	9000 (6910–12,570) ^b^
Neutrophils (N/mm^3^)	1380–7200 ^a^	8475 (3738–12,875)	2870 (2235–4218) ^b^
Lymphocytes (N/mm^3^)	1430–6630 ^a^	3175 (2073–5030)	4450 (3233–6405) ^b^
Platelets (N×1000/mm^3^)	216.0–489.0 ^a^	341.0 (264.8–439.0)	480.0 (356.8–581.5) ^b^
CRP (mg/L)	<20.0 ref. [32,33]	82.7 (51.5–156.4)	4.7 (1.0–13.8) ^b^
PCT (ng/mL)	<0.5 ref. [32,33]	1.57 (0.39–8.17)	0.12 (0.07–0.40) ^b^

SBI: severe bacterial infection; WBC: white blood cells, ; CRP: C-reactive protein; PCT: procalcitonin. ^a^ Santobono-Pausilipon Children’s Hospital laboratory’s reference values; ^b^ *p* < 0.05 versus T0, Wilcoxon test.

**Table 3:** Comparison of lipid profile (mg/dL), HDL class (%) and subfractions (%) in children with SBI at the time of hospitalization (T0) and discharge (T1), and in healthy controls (CTRL).

	CTRL (n = 63)	SBI (n = 63)	
		T0	T1
Total cholesterol	139 (129–161)	102 (87–126) ^a^	145 (120–169) ^b^
LDL-cholesterol	76 (60–92)	45 (30–71) ^a^	74 (59–102) ^b^
HDL-cholesterol	47 (42–54)	28 (20–37) ^a^	29 (22–34) ^a^
Large HDL	33.1 (27.4–37.8)	35.4 (27.6–47.6)	28.6 (21.9–37.4) ^a,b^
Intermediate HDL	47.3 (42.7–50.3)	47.2 (35.1–52.9)	48.9 (41.5–53.0) ^b^
Small HDL	18.4 (14.7–24.0)	17.1 (11.7–22.6)	21.6 (16.1–26.8) ^b^
HDL-1	8.1 (5.9–9.7)	11.1 (7.5–19.2) ^a^	8.2 (4.9–11.5) ^b^
HDL-2	15.3 (12.6–17.8)	14.3 (11.2–16.7)	11.3 (9.0–14.5) ^a,b^
HDL-3	10.2 (7.9–12.3)	9.0 (7.5–11.7)	8.2 (6.5–9.2) ^a,b^
HDL-4	10.6 (9.4–12.3)	11.3 (9.3–13.0)	10.1 (8.2–11.8) ^b^
HDL-5	11.4 (9.9–12.6)	11.9 (9.5–13.9)	12.1 (10.4–14.0)
HDL-6	18.4 (16.4–20.5)	16.0 (11.5–21.2)	19.5 (16.3–22.5) ^b^
HDL-7	6.0 (5.2–6.8)	5.1 (3.7–6.6) ^a^	6.5 (5.2–7.8) ^b^
HDL-8	5.5 (4.4–6.6)	4.5 (3.4–5.9) ^a^	6.0 (5.0–7.2) ^a,b^
HDL-9	4.7 (3.6–5.7)	4.4 (3.2–5.5)	5.3 (4.4–6.9) ^a,b^
HDL-10	8.1 (6.7–11.6)	7.5 (5.0–12.2)	10.3 (7.1–14.7) ^b^

SBI: severe bacterial infection; ^a^ *p* < 0.05 versus CTRL, Mann–Whitney U test; ^b^ *p* < 0.05 versus T0, Wilcoxon test.

## Data Availability

The raw data supporting the conclusions of this article will be made available by the authors on request, as the data are part of an ongoing study.

## Supplementary Materials

The following supporting information can be downloaded at https://www.mdpi.com/article/10.3390/ijms27209300/s1.

## Abbreviations

The following abbreviations are used in this manuscript:

SBI	severe bacterial infection
UTI	urinary tract infection
CRP	C-reactive protein
PCT	procalcitonin
HDL	high-density lipoproteins
MIS-C	multisystem inflammatory syndrome in children
LDL	low-density lipoproteins
CTRL	controls
CoNS	coagulase-negative Staphylococci
IQR	interquartile range
ROC	receiver operating characteristic
BW	body weight
pr_s_	partial Spearman’s correlation coefficient
